# Mechanotransduction and cell fate: from molecular sensors to multicellular self-organization

**DOI:** 10.3389/fcell.2026.1871354

**Published:** 2026-08-06

**Authors:** María del Carmen Estrada Elorza, Mónica Cruz-Lemini, Karina Martínez-Mayorga, Mariana G. Martinez-Garfias, Ana Carolina Gómez-Carrillo, Alfonso Rios-Perez, Julio Granados-Montiel

**Affiliations:** 1 Facultad Mexicana de Medicina, Universidad La Salle México, Mexico City, Mexico; 2 Fetal Cardiology Unit, Hospital de la Santa Creu i Sant Pau, Barcelona, Spain; 3 Instituto de Química, Unidad Mérida, Universidad Nacional Autónoma de México (UNAM), Mérida, Mexico; 4 Departamento de Bioingeniería, Escuela de Ingeniería y Ciencias, Tecnologico de Monterrey, Tlalpan, Mexico City, Mexico; 5 Centre for Craniofacial and Regenerative Biology, Faculty of Dentistry, Oral & Craniofacial Sciences, King’s College London, London, United Kingdom

**Keywords:** cardiac development, cell fate, mechanotransduction, Piezo, regenerative medicine, self-organization, stem cell differentiation, TMC1

## Abstract

Mechanical signals are now recognized as instructive cues that guide cell fate decisions with a precision comparable to classical morphogens. The identification of genetically encoded mechanosensors—including the PIEZO and TMC ion channel families, Transient Receptor Potential channels, and mechanosensitive adhesion complexes—has revealed how cells translate forces into transcriptional programs. In this review we integrate three levels of mechanotransduction biology: the molecular sensors that detect force, the intracellular signaling networks that convert sensing into gene expression, and the multicellular dynamics by which local mechanical interactions drive tissue self-organization. We discuss how substrate stiffness, applied tension, and cell–cell mechanical coupling regulates the differentiation of stem and progenitor cells across diverse lineages, with particular emphasis on the developing cardiovascular system as a paradigmatic mechanobiological organ: primitive blood flow instructs cardiac chamber morphogenesis, and mechanosensitive channels such as PIEZO1 are essential for vascular patterning. We also examine skeletal progenitor commitment and articulate an emerging conceptual distinction—the Regeneration-Specific Mechanosensor hypothesis—proposing that a defined subset of mechanosensors is dispensable during morphogenesis but becomes essential during tissue repair, with TRPA1 as the prototypical example. Structural and computational insights into channel gating, together with engineered mechanical environments for directing stem cell fate, provide a translational bridge toward regenerative therapeutics in cardiovascular and musculoskeletal medicine. Outstanding questions include the hierarchy of mechanosensors during lineage commitment, the mechanical logic of multicellular symmetry breaking, and the translational potential of regeneration-specific mechanosensitive drug targets. We propose that integrating molecular, cellular, and tissue-scale mechanobiology offers a unifying framework for understanding cell fate decisions in both development and regenerative medicine.

## 1 Introduction—mechanical cues as morphogenetic signals

More than a century before molecular biology rendered the question tractable, anatomists and physiologists already suspected that mechanical forces were among the most important sculptors of living form. Wolff’s law of bone remodeling (1892) and D’Arcy Thompson’s On Growth and Form (1917) framed the body not as a passive vessel for chemistry but as an active, force-responsive structure whose final geometry could not be predicted from genes alone. For most of the 20th century, however, the question of how cells actually read those forces remained intractable. Without molecular handles on the sensing apparatus, mechanical biology was relegated to the status of a phenomenological discipline—descriptive, suggestive, but mechanistically opaque.

Two developments transformed this landscape. First, the systematic study of substrate mechanics in cell culture demonstrated that stiffness alone, in the absence of any soluble inductive cue, is sufficient to bias the lineage commitment of mesenchymal stem cells (Engler et al., 2006). Soft substrates resembling brain promoted neurogenic markers, intermediate substrates resembling muscle promoted myogenic markers, and rigid substrates resembling bone promoted osteogenic markers—a remarkable demonstration that the mechanical microenvironment carries instructive information of a fidelity comparable to classical morphogens. Second, the molecular identification of genetically encoded mechanosensors—most prominently the PIEZO ion channel family in 2010 (Coste et al., 2010), followed by the structural characterization of TMC1 in 2022 (Jeong et al., 2022) — provided long-sought molecular handles on the force-sensing apparatus.

These advances converged on a unifying recognition: mechanical signals are not merely permissive scaffolds for chemical signaling, but instructive cues in their own right. They specify lineage, organize tissue architecture, and orchestrate the multicellular dynamics that produce organs of stereotyped form. The central thesis of this review is that mechanotransduction operates simultaneously and inseparably at three scales: a molecular toolkit of genetically encoded sensors; intracellular signaling networks that transduce force into transcription; and multicellular dynamics by which local mechanical interactions generate emergent tissue order. Treating these scales in isolation, as much of the field has historically done, obscures the most consequential biology—which lives in their integration. We pursue this integration with deliberate emphasis on two paradigmatic systems. The developing cardiovascular system offers the most dramatic example of mechanobiological self-organization in vertebrate development: a simple tube transforms into a four-chambered organ under the combined instruction of intrinsic genetic programs and the hemodynamic forces generated by its own early contractions. The skeletal system, with its mechanically loaded growth plates and articular cartilage, offers the most quantitatively tractable example of force-instructed tissue architecture in postnatal life. Together they bracket the developmental and regenerative regimes that this review aims to distinguish.

That distinction motivates a second conceptual contribution. Across multiple mechanosensor families, knockout phenotypes during embryonic development have proved unexpectedly mild, even when the same channels are clearly essential in postnatal physiology. We propose that this pattern is not artifactual but biologically meaningful, and we formalize it here as the Regeneration-Specific Mechanosensor (RSM) hypothesis: a defined subset of mechanosensors is dispensable for morphogenesis but becomes essential for tissue repair, deployed selectively when a tissue must reorganize *de novo* against an altered mechanical landscape. TRPA1 in cartilage provides the prototypical case (Horváth et al., 2016), with parallels emerging in PIEZO2-dependent proprioceptive regulation of skeletal integrity (Assaraf et al., 2020) and TRPV4-dependent cartilage repair. The remainder of the review is organized as follows. Section 2 catalogs the molecular mechanosensor toolkit, including a structural and computational subsection that frames how membrane tension is transduced at atomic resolution. Section 3 examines mechanotransduction in cell fate decisions across pluripotent, mesenchymal, neural, hematopoietic, cardiovascular, and skeletal lineages. Section 4 moves from molecular sensing to multicellular self-organization, with extended attention to cardiac chamber morphogenesis and growth plate columnarization as paradigmatic examples. Section 5 develops the Regeneration-Specific Mechanosensor hypothesis in full. Section 6 surveys therapeutic implications. Section 7 outlines open questions and technological frontiers.

## 2 The molecular mechanosensor toolkit

**FIGURE 1:**
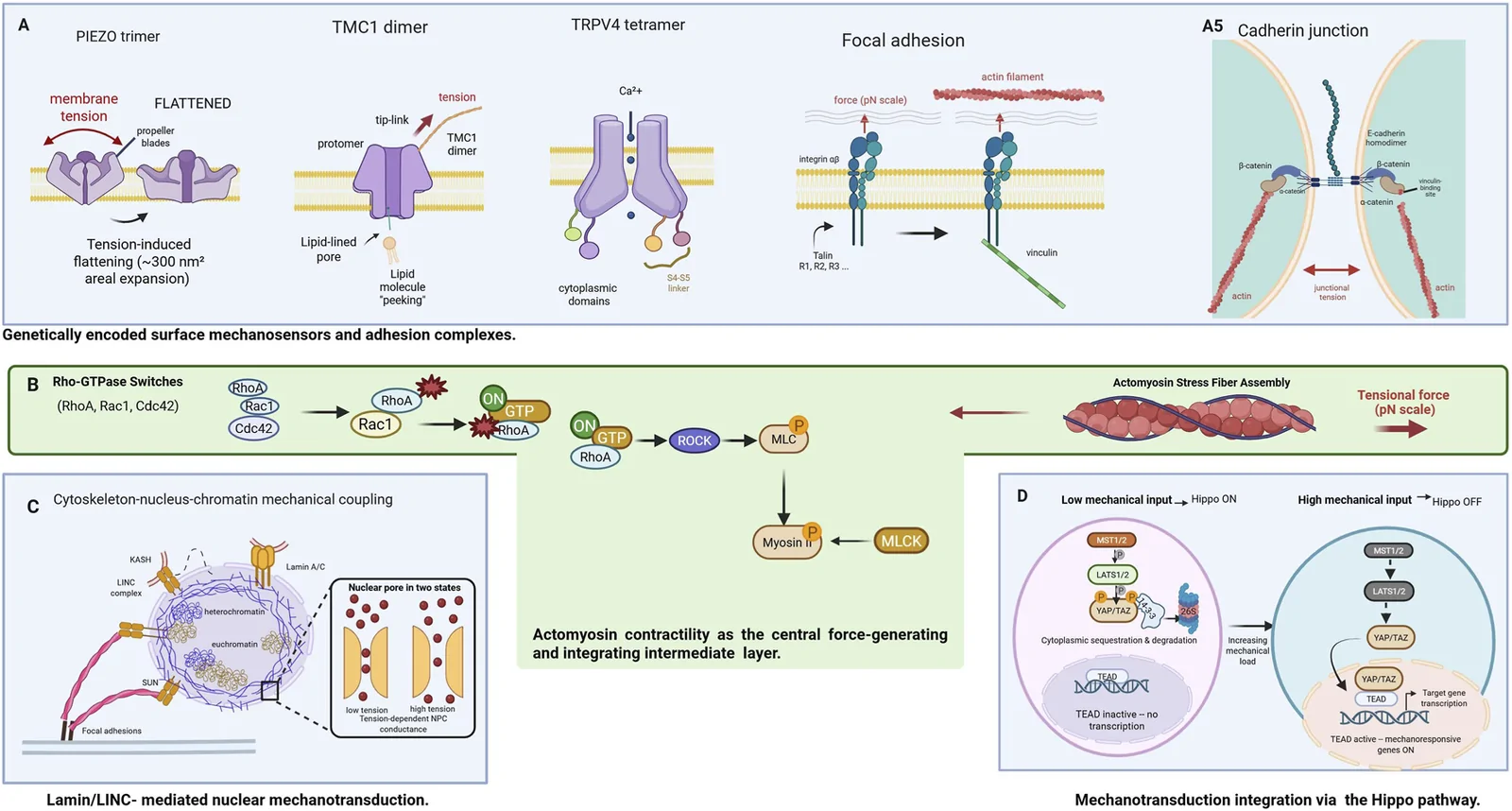
The molecular mechanosensor toolkit. Schematic overview of the principal mechanosensing modules discussed in this review, organized by subcellular tier. **(A)**—Genetically encoded surface mechanosensors and adhesion complexes. (A1) PIEZO trimer in the plasma membrane: membrane tension drives reversible dome-to-flat conformational change with ∼300 nm^2^ areal expansion upon activation (Coste et al., 2010). (A2) TMC1 dimer, with a lipid-lined pore gated by tip-link tension in hair cells; cytoplasmic lipid “peeking” through the protomer is indicated (Jeong et al., 2022). (A3) TRPV4 tetramer transducing osmotic and mechanical input into Ca^2+^ influx *via* the S4–S5 linker. (A4) Integrin-based focal adhesion: piconewton-scale force on the integrin αβ heterodimer drives talin rod (R1, R2, R3…) unfolding, exposing cryptic vinculin-binding sites and reinforcing the actin–integrin clutch. (A5) Cadherin junction: E-cadherin homodimers transmit junctional tension through β-catenin and α-catenin, with the α-catenin vinculin-binding site exposed under load and coupling to the cortical actin network. **(B)**—Cytoskeletal and actomyosin-mediated mechanotransduction: the central force-generating and integrating intermediate layer. Rho-family GTPases (RhoA, Rac1, Cdc42) cycle between inactive GDP-bound and active GTP-bound states. Active RhoA·GTP engages ROCK, which phosphorylates myosin light chain (MLC) both directly and by inhibiting MLC phosphatase; MLCK provides a parallel input. Phosphorylated MLC activates non-muscle myosin II, driving actomyosin stress-fiber assembly and contraction at piconewton-scale tensional force, which feeds back onto adhesion maturation and nuclear mechanotransduction. **(C)**—Lamin/LINC-mediated nuclear mechanotransduction. Cytoskeletal forces are transmitted to the nucleus *via* the LINC complex (SUN–KASH bridge), deforming the lamin A/C meshwork and modulating heterochromatin/euchromatin organization. Inset: tension-dependent nuclear pore complex (NPC) conductance—low nuclear tension restricts and high nuclear tension dilates transport, regulating mechanosensitive transcription-factor access (Elosegui-Artola et al., 2017). **(D)**—Mechanotransduction integration *via* the Hippo pathway. Mechanical inputs from surface sensors **(A)**, the actomyosin cytoskeleton **(B)**, and nuclear deformation **(C)** converge on the Hippo kinase cascade. (D1) Low mechanical input: active MST1/2–LATS1/2 signaling phosphorylates YAP/TAZ, driving 14-3-3-mediated cytoplasmic sequestration and 26S-proteasomal degradation; TEAD is inactive and mechanoresponsive transcription is off. (D2) High mechanical input: the kinase cascade is inactivated, allowing dephosphorylated YAP/TAZ to translocate to the nucleus and bind TEAD to drive mechanoresponsive gene transcription (Dupont et al., 2011).

A working definition of a molecular mechanosensor is a protein whose conformation, activity, or interaction state is altered directly by mechanical force, and whose alteration generates a measurable downstream biochemical signal. Under this definition the eukaryotic mechanosensor inventory now spans ion channels, adhesion complexes, cytoskeletal cross-linkers, and components of the nuclear envelope. Each family employs a distinct biophysical strategy for converting force into signal, and each operates within a characteristic range of stimulus magnitudes and timescales (Figure 1).

### 2.1 PIEZO channels

PIEZO1 and PIEZO2 are the founding members of the genetically defined mechanosensitive ion channel class in vertebrates (Coste et al., 2010). Both adopt a propeller-shaped trimeric architecture with three blades that locally curve the lipid bilayer, generating a domed membrane configuration whose flattening under tension opens a central cation pore (Saotome et al., 2018; Lin et al., 2019; Yang et al., 2022). The lipid bilayer is therefore an intrinsic part of the gating apparatus, not merely a passive medium—a principle now generalized as the force-from-lipid paradigm, supported by the demonstration that purified PIEZO1 is inherently mechanosensitive when reconstituted into lipid bilayers (Syeda et al., 2016). PIEZO1 is broadly expressed in vascular endothelium, urothelium, red blood cells, and bone, where it mediates flow sensing, volume regulation, and skeletal mechanostat function (Sun et al., 2019). PIEZO2 dominates in low-threshold sensory neurons, proprioceptors, and Merkel cells, where it underlies light touch, proprioception, and a non-cell-autonomous role in the maintenance of postnatal skeletal integrity: conditional deletion of Piezo2 in proprioceptive neurons using the PValb-Cre driver produces progressive spinal malalignment and hip dysplasia that phenocopy human PIEZO2 loss-of-function syndromes (Assaraf et al., 2020). Inactivation kinetics of PIEZO1 are themselves mechanosensitive—channels exposed to sustained membrane tension transition from rapid to slow inactivation modes, providing a built-in temporal filter that distinguishes transient from sustained mechanical inputs. This kinetic versatility helps explain how a single channel family can serve such a diverse repertoire of physiological roles. Pharmacologically, the small-molecule agonist Yoda1 stabilizes the open state by binding at the blade–pore interface, while the tarantula peptide GsMTx4 acts as a generic inhibitor of cationic mechanosensitive channels by partitioning into the outer membrane leaflet and locally relieving tension.

### 2.2 TRP channels

Transient Receptor Potential (TRP) channels constitute a structurally and functionally heterogeneous superfamily, several members of which are demonstrably mechanosensitive. TRPV4 has been most thoroughly characterized in this context: in articular chondrocytes, dynamic compressive loading at physiological strain magnitudes activates TRPV4 and triggers an anabolic gene expression program with kinetics indistinguishable from soluble TGF-β stimulation (O’Conor et al., 2014). This finding established TRPV4 as a *bona fide* mechanotransducer in postnatal cartilage homeostasis. That TRPV4 is dispensable for cartilage morphogenesis yet required for the maintenance of cartilage under mechanical load is an early indication that mechanosensor function can be conditional on the mechanical regime—a theme developed in Section 5.

TRPA1 is expressed in chondrocytes, sensory neurons, and a growing list of non-neuronal cell types. Its role as a mechanosensor is more nuanced than that of TRPV4: under standard developmental conditions, Trpa1 knockout mice display no overt skeletal phenotype, and in models of monoiodoacetate-induced osteoarthritis they are indistinguishable from wild-type littermates with respect to cartilage degradation (Horváth et al., 2016). This negative finding, far from indicating biological irrelevance, is one of the cornerstones of the Regeneration-Specific Mechanosensor hypothesis developed in Section 5. TRPM7 contributes to mechanosensitive Mg^2+^ and Ca^2+^ flux in adherent cells, while TRPC1 has been implicated in stretch-activated currents in skeletal muscle and vascular smooth muscle, though its independence as a mechanosensor remains debated. This dissociation between molecular evidence of mechanosensory function and the absence of a developmental phenotype is revisited in Section 5, where we argue that it is not evidence of biological irrelevance but a clue to the conditions under which the channel is actually engaged.

### 2.3 The TMC family

Transmembrane channel-like (TMC) proteins were originally identified as essential components of the mechanotransduction apparatus of inner ear hair cells (Kawashima et al., 2011; Pan et al., 2013). For nearly a decade, their structural organization remained elusive. The cryo-EM structure of TMC1 published in 2022 resolved this gap, revealing a dimeric assembly with each protomer contributing a putative ion-conducting pore positioned at the lipid–protein interface—an architecture compatible with direct gating by membrane tension transmitted through the tip-link complex (Jeong et al., 2022). The TMC pore architecture is reminiscent of the TMEM16 anion channel family, suggesting an ancient evolutionary origin for force-sensitive channels with this structural fold.

More recent expression analyses have identified TMC1 transcripts and proteins in tissues well outside the inner ear, including chondrogenic mesenchymal progenitors. In our own transcriptomic surveys of human mesenchymal stem cells undergoing chondrogenic differentiation in the presence of the small-molecule inducer kartogenin, TMC1 expression rises significantly during commitment, alongside the canonical mechanosensors TRPA1 and COCH. This pattern raises the possibility that the TMC family contributes to mechanotransduction in tissues beyond the auditory system, and constitutes one of the more provocative recent additions to the mechanosensor inventory. Whether the TMC family contributes functionally to mechanotransduction in these non-auditory tissues remains open; as discussed in Section 5, the absence of a non-auditory knockout phenotype under standard evaluation does not by itself settle the question.

### 2.4 Integrin-based mechanosensors and the molecular clutch

Integrin-based focal adhesions function as mechanosensitive nodes that physically couple the extracellular matrix to the actin cytoskeleton. Talin, vinculin, paxillin, and focal adhesion kinase (FAK) form a force-sensitive scaffold whose composition and conformation depend on the magnitude and duration of applied tension. Under load, talin unfolds in a stereotyped manner, exposing cryptic vinculin-binding sites that recruit additional reinforcing proteins—a textbook example of force-induced protein conformational change generating biochemical signal. The molecular clutch model formalizes how the rate of actin retrograde flow, the lifetime of integrin–ligand bonds, and substrate compliance jointly determine whether traction force is efficiently transmitted to the matrix (Chan and Odde, 2008). This framework now underlies much of our understanding of substrate stiffness sensing in motile cells and adhering progenitors.

### 2.5 Cadherin-based cell–cell mechanosensing

E-cadherin and its associated catenin complex transmit and sense forces between neighboring cells. α-Catenin undergoes a force-dependent conformational change that exposes a vinculin-binding site, recruiting actin and reinforcing the adherens junction under tension. p120-catenin and other junctional proteins fine-tune this response. Cadherin-based mechanosensing is essential for collective cell migration, epithelial morphogenesis, and the maintenance of tissue integrity under mechanical stress, and it provides the biophysical substrate for the cell–cell mechanical coupling that drives many of the multicellular self-organization phenomena discussed in Section 4.

### 2.6 Cytoskeletal and actomyosin-mediated mechanotransduction

The membrane channels and adhesion complexes described above detect force at the cell surface, and the nuclear envelope discussed below converts force into changes in genome accessibility. Between these two compartments lies the actomyosin cytoskeleton, which is not a passive conduit but an active and regulated mechanotransducer in its own right. It generates contractile force internally, transmits externally applied force across the cell, and continuously remodels its own architecture in response to the mechanical state it encounters. Treating the cytoskeleton only as plumbing between sensors and nucleus understates its role: much of the decisive mechanotransduction in stem and progenitor cells occurs within this intermediate layer.

#### 2.6.1 Rho-family GTPases as mechanical switches

The Rho-family GTPases—principally RhoA, Rac1, and Cdc42 — act as molecular switches that translate adhesion-derived signals into specific cytoskeletal programs. Cycling between an active GTP-bound and inactive GDP-bound state under the control of guanine-nucleotide exchange factors and GTPase-activating proteins, they convert the engagement and maturation of focal adhesions into directed changes in actin organization and contractility (Huang and Ingber, 2005; Provenzano and Keely, 2011). RhoA and Rac1 are frequently antagonistic: RhoA activity promotes contractile actomyosin bundles and adhesion maturation, while Rac1 favors a branched, protrusive actin network, and constitutive activation of one suppresses the other (Newell-Litwa et al., 2015). This reciprocal regulation allows a cell to adopt distinct mechanical states—contractile *versus* protrusive—in response to substrate stiffness and adhesive context, and it positions the Rho GTPases as a principal decision point in mechanically instructed cell fate.

#### 2.6.2 ROCK and the regulation of actomyosin contractility

The best-characterized RhoA effector in mechanotransduction is Rho-associated kinase (ROCK). Active RhoA engages ROCK, which raises the phosphorylation of regulatory myosin light chain (MLC) both directly and by inhibiting MLC phosphatase; non-muscle myosin II, activated by phosphorylated MLC, then drives contraction of the actin network (Huang and Ingber, 2005; Provenzano and Keely, 2011). MLC is also phosphorylated independently by myosin light-chain kinase (MLCK), so that contractility integrates inputs from more than one upstream pathway. The functional consequences of this module for cell fate are well documented. In mesenchymal stem cells, RhoA and ROCK activity, together with isometric tension in the actin cytoskeleton, are required for mechanically induced osteogenic commitment, and RhoA activation and fluid flow act additively on the osteogenic transcription factor Runx2 while suppressing adipogenic and chondrogenic programs (Arnsdorf et al., 2009). Pharmacological or genetic suppression of contractility—with the ROCK inhibitor Y-27632, MLCK-inhibitory peptides, or the myosin II inhibitor blebbistatin—reproducibly abolishes flow- and stiffness-induced osteogenic differentiation, confirming that actomyosin contractility is necessary, not merely correlated (Yamada et al., 2023). The directionality of the effect is lineage-dependent: in human pluripotent stem cells, increasing actomyosin contractility through constitutively active ROCK or MLCK blocks WNT-driven mesoderm specification, whereas reducing contractility accelerates it (Fort et al., 2026). Contractility is thus an instructive variable whose sign, not only its magnitude, is read differently by different lineages.

#### 2.6.3 Stress fibers and actin remodeling in force transmission

The contractile output of the actomyosin module is organized into stress fibers—bundles of actin filaments crosslinked by α-actinin and interdigitated with non-muscle myosin II—that physically connect focal adhesions to the cell interior. Actin-crosslinking proteins solidify these bundles by limiting the slippage of filaments past one another, which allows myosin-generated force to be transmitted efficiently along the fiber to the adhesion plaque (Katsuta et al., 2024). Stress fibers are more than cables: their assembly at nascent adhesions provides the structural template that licenses focal adhesions to mature compositionally and to remodel the extracellular matrix, a step that a threshold level of tension permits but does not by itself accomplish (Oakes et al., 2012). Continuous actin polymerization, depolymerization, and bundle reorganization therefore allow the cytoskeleton to adjust its stiffness and force-transmitting capacity dynamically as the mechanical environment changes. Because the same architecture that transmits force also gates its delivery to downstream targets, actin remodeling sets the gain of the entire mechanotransduction pathway.

#### 2.6.4 Integration with surface sensors and the nucleus

The actomyosin layer is the hub through which the other mechanotransduction modules communicate. Force sensed at integrin-based adhesions is converted by Rho-GTPase signaling into contractility and stress-fiber assembly; that contractility in turn tensions the adhesions, exposing cryptic binding sites in talin and α-catenin and reinforcing both cell–matrix and cell–cell junctions, so that adhesion sensing and cytoskeletal contraction operate as a single mechanically coupled feedback loop. The same contractile network determines the activity of the nuclear effectors discussed in the following subsections. Cytoskeletal tension and actomyosin contractility are upstream requirements for the nuclear accumulation of YAP and TAZ, and contractile force transmitted through the LINC complex deforms the nuclear lamina and alters chromatin organization and nuclear-pore conductance (Discher et al., 2017). The actomyosin cytoskeleton is, in this sense, the integrating element of mechanotransduction: it is where force generated internally and force applied externally are combined into the single mechanical signal that the nucleus ultimately reads.

### 2.7 Lamin/LINC-mediated nuclear mechanotransduction

The nuclear envelope is no longer regarded as a passive container for the genome but as an active mechanosensitive organelle. The nuclear lamina, composed primarily of A-type and B-type lamins, provides mechanical stiffness to the nucleus, while the LINC (Linker of Nucleoskeleton and Cytoskeleton) complex physically couples cytoskeletal forces to the nuclear interior. Mechanical deformation of the nucleus alters chromatin organization, opens nuclear pore complexes, and modulates the accessibility of specific genomic loci to transcription factors. Lamin A/C levels themselves scale with tissue stiffness and feedback on cell fate decisions, completing a remarkable mechanochemical loop in which the nucleus is both target and regulator of cellular force sensing.

### 2.8 The Hippo pathway and YAP/TAZ

The transcriptional co-activators YAP and TAZ are the most extensively characterized nuclear effectors of cellular mechanics. On stiff substrates and under high cytoskeletal tension, YAP/TAZ accumulate in the nucleus and partner with TEAD transcription factors to drive proliferation, lineage commitment, and tissue growth programs; on soft substrates and under low tension, YAP/TAZ are sequestered and degraded in the cytoplasm (Dupont et al., 2011). Their localization is governed by an integrated input from substrate stiffness, cell shape, cytoskeletal contractility, and intercellular tension transmitted through cadherin junctions, making them a near-canonical mechanical rheostat (Aragona et al., 2013; Dupont et al., 2011). The biophysical chain from membrane to nucleus involves Rho-family GTPase activation, actomyosin contractility, focal adhesion maturation, and direct nuclear deformation that opens nuclear pore complexes to facilitate YAP/TAZ import.

### 2.9 Structural and computational insights into mechanosensor gating

Cryo-electron microscopy and molecular dynamics simulation have together transformed our atomistic understanding of how membrane tension opens mechanosensitive channels. The trimeric architecture of PIEZO1 — three propeller blades bowing the surrounding lipid bilayer into a curved dome—provides a geometric mechanism by which membrane tension is converted into pore opening: tension flattens the dome and the resulting conformational change widens the central conduction pathway (Lin et al., 2019). All-atom molecular dynamics simulations performed in tensioned bilayers have visualized this flattening on physiologically relevant timescales and have identified specific lipid species, particularly phosphatidylinositol bisphosphates, that allosterically modulate gating efficiency. The 2022 cryo-EM structure of TMC1 revealed a dimeric channel with each protomer contributing a pore positioned at the lipid–protein interface, an architecture compatible with direct lipid-mediated gating (Jeong et al., 2022). Comparative analysis with the TMEM16 family suggests evolutionary conservation of the fold and supports the hypothesis that membrane tension is sensed locally at the periphery of each protomer rather than at a single bilayer-spanning sensor. The TRP channel family adopts a tetrameric architecture in which the S4–S5 linker couples voltage- and ligand-sensing modules to the pore; for mechanosensitive members such as TRPV4, molecular dynamics has identified specific membrane-facing residues whose perturbation by tension biases the channel toward the open state.

Computational docking studies of small-molecule modulators—Yoda1 at the PIEZO1 blade–pore interface, GsMTx4 partitioning into the outer leaflet, GSK205 binding TRPV4 — provide structural rationales for the pharmacological repertoire that will become essential as mechanosensitive drug targets advance toward clinical application (Section 6). Importantly, the protein–lipid interface is increasingly recognized as the primary control surface for mechanosensitive channel pharmacology, suggesting that lipid-modulating compounds may complement direct channel agonists and antagonists in future therapeutic strategies. These structural and computational advances are not peripheral to mechanobiology; they constitute the molecular grammar without which mechanosensation cannot be rationalized at the level required by modern translational programs.

## 3 Mechanotransduction in cell fate decisions

**FIGURE 2:**
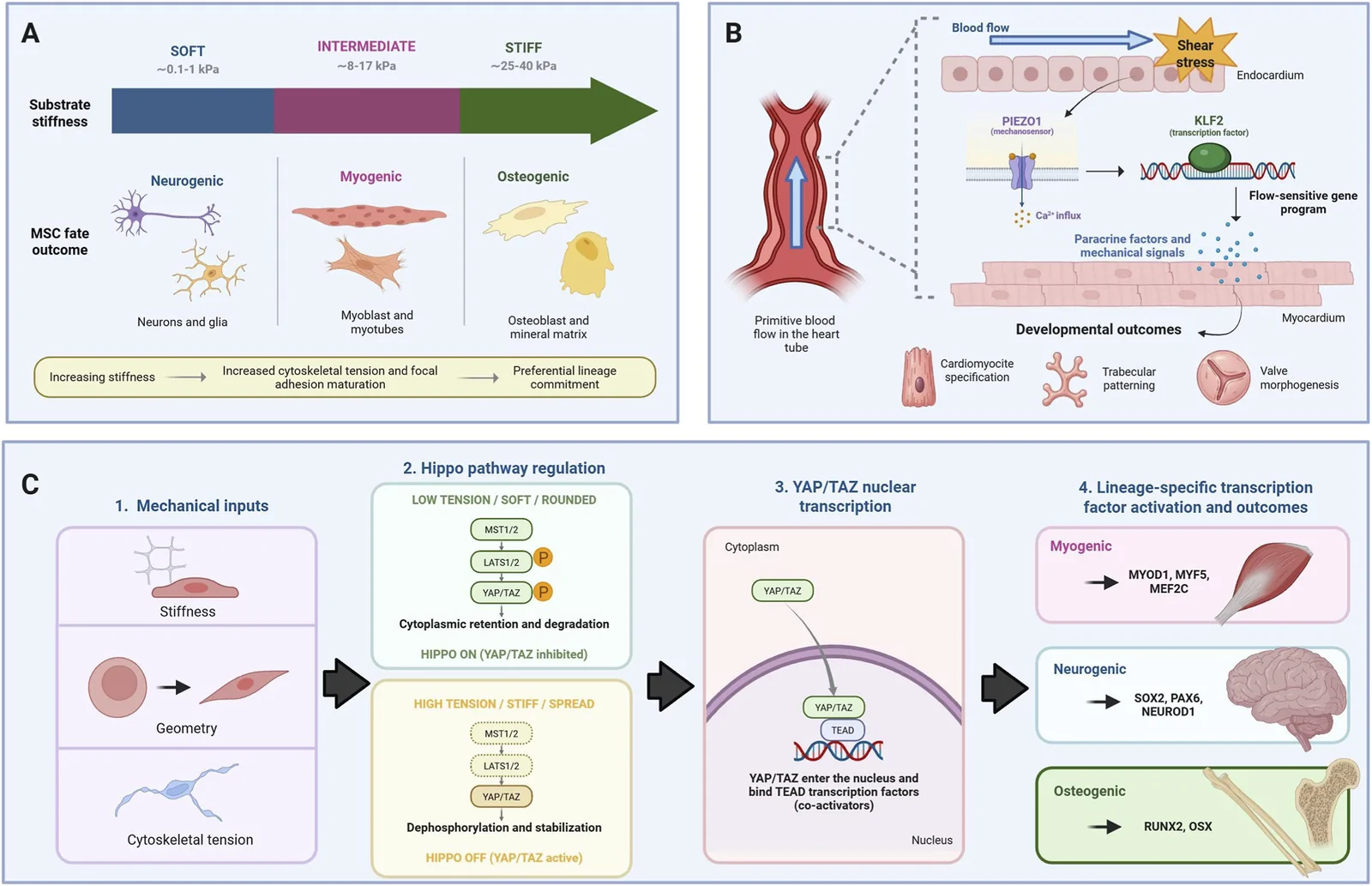
Mechanical cues in cell fate decisions. **(A)** Substrate stiffness biases mesenchymal stem cell lineage commitment, with neurogenic, myogenic, and osteogenic outcomes correlating with substrates of brain-, muscle-, and bone-like elastic moduli respectively (after Engler et al., 2006), overlaid with the dominant mechanosensor families engaged at each stiffness regime. **(B)** Cardiovascular subpanel: primitive blood flow in the embryonic heart tube generates shear stress on the endocardium that instructs cardiomyocyte specification, trabecular patterning, and valve morphogenesis, with PIEZO1 and KLF2 as principal molecular mediators. **(C)** Hippo pathway as integrator: stiffness, geometry, and cytoskeletal tension converge on YAP/TAZ nuclear translocation, which in turn drives lineage-specific transcription factor activation.

Having cataloged the molecular toolkit, we now consider how mechanical signals are read by stem and progenitor cells across major lineages. The unifying observation is that mechanical inputs are not merely permissive—they are instructive, and they engage the same cellular decision-making machinery that responds to soluble morphogens. Across every lineage examined to date, substrate mechanics, applied tension, or intercellular force coupling is sufficient to bias commitment in the absence of supplementary chemical cues, and necessary to permit normal commitment in the presence of those cues (Figure 2).

### 3.1 Pluripotent stem cell mechanics

Embryonic and induced pluripotent stem cells are mechanically responsive from the earliest stages of culture, and their mechanical environment influences the balance between self-renewal and differentiation as directly as soluble signaling does. The classic demonstration of this sensitivity used single-cell magnetic twisting cytometry to show that brief, localized stress applied to the surface of mouse embryonic stem cells is sufficient to alter their gene expression, and that this responsiveness is itself developmentally regulated, declining as cells exit the pluripotent state (Chowdhury et al., 2010). Pluripotent cells are therefore not mechanically inert substrates awaiting chemical instruction; they actively interpret force.

Several distinct mechanical variables contribute to this interpretation. Substrate compliance biases the trajectory of differentiation, with compliant substrates favoring particular lineage outcomes even when soluble inductive factors are held constant; compliant culture surfaces, for example, promote efficient neuronal differentiation of human pluripotent stem cells by limiting YAP nuclear localization, so that the mechanical and biochemical inputs converge on the same nuclear effector (Musah et al., 2014). Colony geometry and local cell density impose additional mechanical constraints: cells at a colony edge experience a different tension and adhesion context than cells in the interior, and these positional differences correlate with heterogeneity in pluripotency-factor expression and with the location at which differentiation initiates. Applied strain and the stiffness of the culture surface together shape the self-renewal-versus-differentiation decision through the adhesion, actomyosin, and YAP/TAZ machinery described in Section 2.

The actomyosin machinery introduced in Section 2.6 operates with particular force in pluripotent stem cell fate decisions. Increasing actomyosin contractility through constitutively active ROCK or MLCK blocks WNT-driven mesoderm specification in human pluripotent stem cells, whereas reducing contractility accelerates it (Fort et al., 2026). The directionality of this effect is opposite to what is seen in mesenchymal commitment, where higher contractility favors osteogenic over adipogenic fate (Arnsdorf et al., 2009), reinforcing the principle that contractility is read differently by different lineages rather than acting as a unidirectional permissive signal. Geometric confinement of pluripotent colonies onto micropatterned substrates is sufficient to produce reproducible spatial patterns of germ-layer specification, with cells at colony edges adopting different fates than interior cells under identical soluble cues (Warmflash et al., 2014), demonstrating that mechanical context is instructive at the level of self-organized tissue patterning, not only of single-cell fate. Conversely, the ROCK inhibitor Y-27632 — originally introduced as a survival adjunct to prevent dissociation-induced apoptosis of human pluripotent stem cells (Watanabe et al., 2007) — has been reinterpreted as a deliberate mechanical input that shapes downstream lineage outcomes when applied during specific differentiation windows, illustrating that an intervention originally framed as cytoprotective is in fact mechanobiological.

These observations have a practical corollary. Because the mechanical environment is instructive, it can be engineered deliberately, and mechanical priming—the controlled pre-exposure of pluripotent cells to defined stiffness, geometry, or strain—has become a recognized strategy for improving the efficiency and reproducibility of directed differentiation. The increasing use of micropatterned substrates, soft-substrate culture, and geometrically defined colonies in pluripotent stem cell laboratories reflects this shift from treating mechanics as an uncontrolled background variable to treating it as a designed input. The mechanical sensitivity of pluripotent cells thus connects directly to the engineered-niche approaches discussed in Section 6.

### 3.2 Mesenchymal stem cell lineage commitment

The Engler study remains the founding demonstration that substrate stiffness alone is sufficient to bias mesenchymal stem cell lineage commitment (Engler et al., 2006). Subsequent work integrated this finding with the YAP/TAZ pathway, establishing nuclear translocation of these co-activators as a near-universal readout of stiffness sensing in MSCs (Dupont et al., 2011). The chondrogenic program is particularly informative because it is associated with a low-stiffness, three-dimensional environment and a distinct transcriptional signature including the SERPIN family members SERPINA9 and SERPINB2 as lineage markers (Granados-Montiel et al., 2021). Recent transcriptomic surveys of MSCs undergoing chondrogenic commitment have revealed coordinated upregulation of mechanosensors of the TRP and TMC families, alongside marked downregulation of the dipeptidyl peptidase 4 (DPP4) axis, suggesting that the mechanical environment of cartilage commitment selects for a specific mechanosensor inventory rather than a generic one.

### 3.3 Neural fate decisions

Neural progenitor cells display a soft-substrate preference that is among the most reproducible findings in cellular mechanobiology. For rodent neural stem cells, substrates with elastic moduli in the range of brain parenchyma—on the order of 0.1 to 1 kPa—promote neurogenic differentiation, whereas stiffer substrates suppress neurogenesis and favor glial commitment (Saha et al., 2008). This stiffness sensitivity is not a fixed property of the cell but is gated in time: dynamic-stiffness culture platforms that allow the substrate to be softened or stiffened at defined moments have identified a narrow window, within roughly the first one to 2 days of differentiation, during which lineage commitment is maximally responsive to mechanical input and after which it becomes insensitive to further stiffness change (Rammensee et al., 2017).

The molecular mediators of this response are increasingly well defined and connect directly to the machinery of Section 2. Soft substrates favor low actomyosin tension and cytoskeletal relaxation, conditions under which YAP is retained in the cytoplasm; stiff substrates raise tension and drive YAP into the nucleus, where it suppresses neurogenesis, in part by sequestering β-catenin and limiting β-catenin-dependent transcription. Manipulating YAP within the mechanosensitive window is sufficient to override the substrate cue—YAP overexpression suppresses neurogenesis on soft substrates and YAP silencing enhances it on stiff ones—establishing YAP as both necessary and sufficient for stiffness-directed neural fate (Rammensee et al., 2017). Upstream of YAP, Rho-family GTPase-regulated contractile force links substrate stiffness to the nuclear readout, and mechanosensitive channels contribute to the same pathway: PIEZO1 activity influences YAP localization in human neural stem and progenitor cells and biases their lineage choice (Pathak et al., 2014). It is worth noting that the direction of the stiffness effect is not universal—human neural progenitors have been reported to favor neuronal differentiation on stiffer substrates than rodent cells do—which indicates that the absolute stiffness setpoint is species- and context-dependent even though the involvement of the YAP–contractility axis is conserved.

Mechanical instruction of neural fate extends beyond stiffness to the geometry of the substrate. Anisotropic features—patterned grooves, aligned fibers, and microchannels—direct the orientation of neurite outgrowth, providing a mechanical analog of the chemical guidance cues that pattern axon trajectories *in vivo*. Substrate topography therefore acts alongside substrate compliance as an instructive input, shaping not only whether a progenitor becomes a neuron but how that neuron is spatially organized.

Substrate elasticity, however, captures only one dimension of the mechanical environment that neural progenitors experience. Brain parenchyma is not a purely elastic solid but a viscoelastic material that dissipates stress over time, and the recognition that this stress-relaxation behavior is itself an instructive cue represents one of the most consequential recent advances in neural mechanobiology. On two-dimensional substrates engineered to relax stress at tunable rates while holding initial stiffness constant, rat neural stem cells shift their lineage output toward astrocytic differentiation as the rate of stress relaxation increases, demonstrating that the time-dependent component of the mechanical response carries lineage information independent of stiffness (Qiao et al., 2024). Mechanistically, faster stress relaxation slows intracellular actin flow, promotes the formation of phosphorylated focal adhesion kinase (pFAK) adhesion clutches, and drives cyclic, high-frequency activation of RhoA; pharmacological inhibition of myosin II or of FAK reverts the lineage distribution toward that observed on elastic substrates, identifying a RhoA–actomyosin–FAK motor-clutch axis as the transducer of viscoelastic input (Qiao et al., 2024). Because viscoelastic properties change in development, aging, and disease, this stress-relaxation dimension is not a laboratory curiosity but a physiologically and pathologically regulated variable, and a contemporary account of neural fate mechanics must treat it alongside stiffness rather than as a refinement of it.

### 3.4 Hematopoietic niche mechanics

The bone marrow is a mechanically heterogeneous tissue, and hematopoietic stem and progenitor cells (HSPCs) are now recognized as mechanically responsive participants in it rather than passive occupants. The marrow contains sub-niches of markedly different stiffness—soft marrow adipose tissue, an intermediate perivascular compartment, and a substantially stiffer endosteal region—and engineered substrates that reproduce these moduli show that the mechanical environment is itself instructive for hematopoietic fate. Stiffer matrices favor the preservation of long-term repopulating hematopoietic stem cells in a relatively quiescent state, whereas softer matrices support the maintenance of multipotent progenitors, indicating that the endosteal and perivascular niches are not mechanically interchangeable (Choi and Harley, 2017). Substrate elasticity alone can drive the expansion of hematopoietic stem and progenitor populations, which has direct relevance to *ex vivo* expansion protocols.

At the molecular level, the mechanosensitive channel PIEZO1 is the best-characterized force sensor in this system. PIEZO1 is expressed in HSPCs and across the cellular constituents of the niche, and it contributes to lineage decisions, with experimental evidence implicating PIEZO1 activity in erythroid and megakaryocytic output; transient pharmacological activation of PIEZO1 has been used to facilitate the *ex vivo* expansion of hematopoietic stem cells (Wang et al., 2026). The megakaryocytic lineage illustrates how tightly this sensing is tuned to matrix mechanics: under physiological marrow stiffness, moderate PIEZO1 activity permits normal megakaryocyte maturation and platelet production, whereas the pathological stiffening characteristic of myelofibrosis drives PIEZO1 overexpression and impairs proplatelet formation, contributing to the aberrant megakaryopoiesis of that disease (Abbonante et al., 2024). The endosteal niche, with its higher stiffness and intermittent compressive loading, and the perivascular niche, with its softer matrix and pulsatile shear from blood flow, thus impose distinct mechanical regimes whose disruption is associated with hematopoietic disease.

The hematopoietic niche also offers an instructive example of mechanically driven repair. Following irradiation injury, the structural disruption of the marrow alters its mechanical environment, and PIEZO1 expression is upregulated in resident bone marrow macrophages in response; this PIEZO1-dependent mechanosensing promotes the regeneration of the sinusoidal vasculature on which hematopoietic reconstitution depends (Zhang et al., 2022). A channel that operates at moderate activity during homeostasis is therefore recruited more prominently when the tissue must rebuild against an altered mechanical landscape—a pattern that prefigures the developmental-versus-regenerative distinction formalized in Section 5.

Beyond the static stiffness of the marrow sub-niches, hematopoietic stem cells are also exposed to the fluid shear stress of blood flow, and recent work has established this hemodynamic force as a determinant of HSC fate with direct relevance to aging. PIEZO1 senses shear stress in the circulation and converts it into ion currents and calcium influx in both mouse and human HSCs, driving their proliferation and biasing differentiation toward the myeloid lineage through downstream JAM3-and CAPN2-dependent signaling (Shang et al., 2026). Because chronic inflammation—a hallmark of aging—is accompanied by altered hemodynamics, this shear-sensing axis provides a mechanistic link between mechanical force and inflammation-induced HSC aging, the myeloid-biased, self-renewal-depleted state characteristic of the aged hematopoietic system; consistent with a causal role, the peptidic PIEZO1 antagonist GsMTx4 attenuates inflammation-induced HSC aging in mice by dampening PIEZO1-dependent HSC activation (Shang et al., 2026). This shear-stress framework reframes hematopoietic niche mechanics: the niche is defined not only by the stiffness of its compartments but by the dynamic fluid forces traversing them, and mechanosensitive HSC aging emerges as a tractable target for intervention.

### 3.5 Cardiovascular lineage commitment and fetal heart morphogenesis

The cardiovascular system is the paradigmatic mechanobiological organ, and its development cannot be understood without explicit attention to mechanical forces. The earliest demonstration of this principle came from chick embryo studies showing that primitive blood flow is not merely a downstream consequence of cardiac contraction but an essential instructive signal for cardiac chamber morphogenesis: when intracardiac flow patterns are surgically perturbed before chamber formation, the resulting heart is structurally abnormal in ways that cannot be explained by genetic or biochemical defects (Hogers et al., 1997). The implication is profound—the heart shapes itself in part by sensing the very fluid forces it generates.

At the molecular level, PIEZO1 has emerged as an essential regulator of vascular development. Endothelial-specific deletion of Piezo1 produces embryonic lethality with severe vascular patterning defects, establishing this channel as a non-redundant flow sensor in the developing vasculature (Ranade et al., 2014). Klf2, a flow-responsive transcription factor, integrates shear stress signals into the endothelial transcriptional program and is required for proper valve morphogenesis. Cardiomyocyte differentiation and maturation are likewise mechanosensitive: cyclic strain applied to differentiating pluripotent stem cells accelerates cardiomyocyte specification, increases expression of mature sarcomeric components, and improves contractile organization.

From the perspective of clinical fetal cardiology, mechanical perturbations during human cardiac development reveal mechanobiological principles that would otherwise remain hidden. Twin-to-twin transfusion syndrome, in which monochorionic twins experience grossly altered hemodynamic loading, produces stereotyped cardiac remodeling in the recipient twin that depends quantitatively on the magnitude of the volume challenge. Congenital heart disease phenotypes associated with altered intracardiac flow—hypoplastic left heart syndrome being the canonical example—illustrate how the developing heart’s structural fate is conditional on its hemodynamic environment. Trabecular *versus* compact myocardium patterning, regional valve thickening, and great vessel caliber are all explicable in part as mechanobiological self-organization phenomena driven by intracardiac shear and wall stress.

Together these findings establish the cardiovascular system as the most quantitatively tractable example of mechanically instructed lineage commitment and tissue patterning in vertebrate development, and they provide the experimental foundation for the mechanobiological framework developed in Section 4.

### 3.6 Skeletal progenitor commitment

The skeletal system is the second paradigmatic example of mechanically instructed cell fate. Mesenchymal progenitors at the limb bud and within the periosteum face a binary fate choice between osteogenic and chondrogenic commitment, and the mechanical microenvironment is among the most powerful instructive cues governing this decision. Compressive loading, low oxygen tension, and a pericellular matrix dominated by type II collagen and aggrecan favor chondrogenic specification; tensile loading, higher oxygen tension, and a periosteal matrix dominated by type I collagen favor osteogenic specification. The growth plate, in which proliferating chondrocytes columnarize and hypertrophy under axial compression before being replaced by bone, provides the most quantitatively analyzable example of force-instructed tissue architecture in postnatal mammalian biology, and it is examined in detail in Section 4. Articular cartilage, with its sustained compressive loading, mechanosensitive expression of TRPV4 and other channels, and notoriously limited regenerative capacity, is the canonical mechanosensitive postnatal tissue and the most relevant target for the regeneration-specific framework developed below.

## 4 From molecular sensing to multicellular self-organization

The most compelling argument for an integrated mechanobiology is that the same molecular sensors that direct the fate of an individual cell also organize the collective behavior of cell populations. Force is among the few signals that can be transmitted instantaneously across many cell diameters through stiff cytoskeletal and matrix elements, and this propagation property makes mechanics uniquely suited to coordinate multicellular dynamics across length scales that exceed the diffusion range of soluble morphogens.

### 4.1 Self-organization principles

Self-organization in biology refers to the emergence of stereotyped large-scale order from local interactions among components, without an external blueprint or master controller. The recent flourishing of gastruloid, embryoid, and organoid systems has shown that surprisingly small numbers of pluripotent or progenitor cells, given an appropriate three-dimensional and biochemical environment, can spontaneously organize into structures recapitulating axial patterning, germ layer specification, and even crude organ morphology (Simunovic and Brivanlou, 2017). Mechanics enters these systems at multiple levels: the geometry and stiffness of the culture vessel bias initial symmetry breaking; cell–cell mechanical coupling propagates polarization signals across the aggregate; and the emergent tissue architecture imposes new mechanical constraints that feedback on subsequent commitment decisions.

### 4.2 Symmetry breaking through mechanics

The transition from a spherically symmetric aggregate to a polarized tissue with an anteroposterior axis is among the most fundamental events in animal development, and it is increasingly recognized as a mechanobiological problem. Local accumulation of actomyosin contractility at one pole of an aggregate generates a tension asymmetry that can bias the localization of polarity proteins, the orientation of cell divisions, and the deposition of extracellular matrix components. In gastruloid systems, the spontaneous emergence of a single anterior–posterior axis from an initially symmetric aggregate has been linked to coupled mechanical and Wnt-signaling feedback loops, providing a tractable experimental setting in which to dissect how mechanics participates in symmetry breaking. Recent work has begun to resolve which physical process executes this symmetry breaking. Using synthetic signal-recording gene circuits to trace the history of Wnt activity in mouse gastruloids, McNamara et al. (2024) showed that an initially patchy, heterogeneous distribution of Wnt signaling is consolidated into a single posterior pole not by a long-range reaction–diffusion instability but by cell sorting—the active mechanical rearrangement of cells—with the earliest detectable asymmetry traceable to prior heterogeneity in Nodal signaling. Symmetry breaking in these aggregates is therefore a mechanically executed event, in which cell movement converts a diffuse signaling bias into a discrete axis. The general principle is that mechanical fields, like chemical fields, can be unstable in regimes that favor the spontaneous amplification of small fluctuations into stereotyped patterns.

### 4.3 Growth plate columnar organization

The mammalian growth plate is among the most striking examples of stereotyped multicellular self-organization in postnatal life. Within a few hundred microns, mesenchymal progenitors give rise to a layered architecture of resting, proliferative, prehypertrophic, and hypertrophic chondrocytes, with the proliferative zone organized into columns of cells aligned along the axis of growth. The columnar arrangement is not imposed by an external scaffold; it emerges from the integration of intrinsic genetic programs (the Indian hedgehog–parathyroid hormone-related protein feedback loop being central) with the compressive mechanical environment of the loaded long bone. Disruption of either input—genetic or mechanical—disrupts the columnar architecture in characteristic ways, and the resulting growth plate dysplasias provide a phenomenologically rich model system for testing mechanobiological hypotheses.

### 4.4 Cardiac chamber morphogenesis as a mechanobiological self-organization paradigm

The embryonic heart provides the most dramatic example of mechanobiological self-organization in vertebrate development. A simple cardiac tube transforms into a four-chambered organ over a period of days, under the combined instruction of intrinsic genetic programs and the hemodynamic forces generated by the heart’s own early contractions. The looping of the heart tube, the ballooning of the chambers, the trabeculation of the ventricular wall, and the morphogenesis of the cardiac valves are all conditional on appropriate intracardiac flow patterns. When flow is experimentally altered before any of these events, the resulting morphology is altered in stereotyped ways that cannot be explained by changes in gene expression alone.

The trabecular myocardium provides a particularly clear example. Trabeculae are not laid down by a separate developmental program; they emerge from the compact ventricular wall under the influence of intracardiac shear stress acting on the endocardial surface. The flow-responsive transcription factor Klf2 is enriched at sites of trabeculation, and its perturbation produces stereotyped trabeculation defects. Valve development is similarly mechanosensitive: oscillatory shear at the developing atrioventricular canal triggers endocardial-to-mesenchymal transition and cushion formation, and the cushion subsequently remodels under sustained flow to produce the mature valve leaflet. Recent work has clarified how this remodeling is mechanically encoded. Wang et al. (2023) decoupled shear and hydrostatic stress and showed that they are read out through YAP in distinct valve cell populations: low oscillatory shear promotes YAP nuclear translocation in valvular endothelial cells while high unidirectional shear restricts it, and hydrostatic compression activates YAP in valvular interstitial cells while tension deactivates it. *In vivo* restriction of flow produced hypoplastic, malformed atrioventricular valves with suppressed YAP, demonstrating that local stresses, transduced through YAP, size and shape the leaflet without a genetically prescribed timing mechanism. Each of these phenomena is a mechanobiological self-organization process—local mechanical interactions between flow, endocardium, and myocardium generating stereotyped large-scale architecture.

### 4.5 Tissue engineering implications

If mechanical signals are instructive for cell fate and self-organization, then the design of regenerative tissue constructs must explicitly engineer the mechanical environment, not merely the chemical one. Hydrogels with graded stiffness, anisotropic fiber alignment, and tunable degradation kinetics now permit experimental control over the mechanical microenvironment with a precision approaching that of biochemical control. Bioprinting permits the spatial deposition of cells within mechanically defined matrices, and organ-on-chip platforms permit the application of physiologically relevant flow, strain, and pressure to engineered tissue constructs. A recent body of work has converged bioprinting and organ-on-chip technologies specifically around mechanobiological control: the combination allows cells and organoids to be deposited within hydrogels whose stiffness and architecture are individually tunable, and integrated microfluidic perfusion to apply defined flow and strain, so that the mechanical microenvironment can be reconstructed with a precision that earlier static culture could not achieve (Mierke, 2024). The practical consequence is that engineered niches can now be designed to evolve mechanically during repair rather than presenting a fixed substrate. The most successful regenerative engineering programs are those in which mechanical and biochemical design are integrated from the outset, and the failures are concentrated in approaches that treat mechanics as a downstream variable to be optimized after biochemistry.

### 4.6 Cell–ECM feedback loops

Mechanotransduction is fundamentally a feedback phenomenon. Cells sense the mechanical properties of their matrix and respond by depositing, remodeling, or degrading matrix components; these matrix changes alter the local mechanical environment, which in turn alters cellular behavior. Such mechanochemical feedback loops are the substrate for many of the most important mechanobiological diseases, from fibrosis (in which feedback amplification produces pathological stiffening) to osteoarthritis (in which feedback collapse produces pathological softening) to cancer metastasis (in which feedback rewiring produces pathological invasion). The fibrotic case has been resolved into an explicit molecular circuit: Niu et al. (2022) identified a mutually reinforcing positive feedback loop between integrin β1 and the PIEZO1 channel that, through downstream Ca2+ and YAP signaling, forms a bistable switch—matrix stiffening amplifies sensor activity, which drives further matrix stiffening, eventually locking cardiac fibroblasts into an activated state. Critically, simultaneously interrupting the feedback loop and lowering matrix stiffness reversed markers of the activated phenotype, indicating that the bistability is, in principle, a therapeutic target rather than a one-way trajectory. They also explain why static analyses of mechanical environments tend to underpredict the magnitude of cellular responses observed *in vivo*: the feedback gain in many tissues is high enough that small perturbations are rapidly amplified into substantial changes in tissue mechanics.

Taken together, Sections 2 through 4 surface a pattern that the preceding discussion has noted case by case but not yet drawn together. Several mechanosensors that are expressed in a tissue, transcriptionally regulated during its cell fate decisions, and biochemically force-responsive *in vitro* nonetheless prove dispensable when removed constitutively and evaluated under standard developmental and homeostatic conditions—TRPA1 in cartilage, TRPV4 for cartilage morphogenesis, the TMC family outside the inner ear, TRPC1 across multiple tissues, and PIEZO2 in the skeletal lineages themselves. This recurs alongside the opposite observation, equally well documented in these sections, that other mechanosensors are strictly required for morphogenesis and are embryonic-lethal on loss. The coexistence of these two groups, and in particular the mismatch between the molecular prominence of the first group and its phenotypic silence, is the empirical problem that Section 5 addresses. We take it up not as a collection of isolated negative results but as a structured pattern in need of an organizing interpretation.

## 5 Developmental *versus* regenerative mechanotransduction: the regeneration-specific mechanosensor (RSM) hypothesis

The preceding sections have surveyed the molecular toolkit of mechanosensation, the mechanically instructed lineage commitment of stem and progenitor cells, and the multicellular self-organization phenomena that emerge when force-sensing operates across scales. We now articulate a conceptual framework that has been implicit in the literature for some years but has not, to our knowledge, been formalized: the Regeneration-Specific Mechanosensor (RSM) hypothesis. We propose this framework explicitly because the experimental implications are substantial and the absence of an organizing concept has, in our view, slowed the field’s progress on a coherent set of observations.

### 5.1 The knockout paradox

The biological literature on mechanosensitive ion channels contains a recurrent and underappreciated pattern: knockout phenotypes during embryonic development are frequently milder than would be predicted from the channels’ postnatal physiological roles, their evolutionary conservation, or their tissue-specific expression patterns. This discordance is most conspicuous among mechanosensors whose adult function is demonstrably non-redundant yet whose constitutive loss produces normal development and often normal homeostasis. Before articulating our interpretive framework, we catalog the principal cases and examine the structural features they share.

TRPA1 provides the cleanest prototypical example. Trpa1-null mice develop normally, produce skeletons indistinguishable from wild-type at every developmental stage examined, and reach adulthood without musculoskeletal phenotype (Horváth et al., 2016). Remarkably, even in experimental osteoarthritis induced by intra-articular monoiodoacetate—a model in which cartilage degradation is severe and reproducible—Trpa1-null animals are indistinguishable from wild-type littermates in histological grading, matrix loss, and pain-related behavior. The channel is expressed in chondrocytes, is upregulated during chondrogenic commitment in our own transcriptomic surveys of kartogenin-induced mesenchymal commitment (GEO accessions GSE325268 and GSE325419; see Data Availability Statement), and produces measurable chondrocyte responses *in vitro* under pharmacological modulation, yet its genetic ablation produces no phenotype in either development or induced disease under this paradigm. Under the classical interpretive framework, the expected conclusion is that TRPA1 is not a biologically important mechanosensor in cartilage. We will argue that this conclusion is premature.

TRPV4 occupies an intermediate position. Trpv4-null mice display mild and largely tissue-specific developmental phenotypes, but their articular cartilage shows age-progressive degeneration and a blunted anabolic response to dynamic compressive loading (O’Conor et al., 2014; Clark et al., 2010). The channel is not developmentally essential, but it is required for the maintenance of cartilage homeostasis under physiological mechanical challenge. TRPV4 thus establishes that mechanosensor function can be conditional on the mechanical regime in which the tissue operates—a principle that the RSM framework will generalize. The pattern extends well beyond these two examples. TMC1 and TMC2 knockouts produce deafness but leave other tissues in which these channels are expressed—including chondrogenic mesenchymal progenitors—apparently unaffected under standard evaluation. COCH mutations cause progressive late-onset hearing loss with vestibular dysfunction despite expression of the protein throughout cochlear development (Robertson et al., 1998), indicating a role in maintenance rather than morphogenesis. Trpc1-null mice display a notably mild phenotype despite the channel’s longstanding identification as a stretch-activated conduit in multiple tissues (Maroto et al., 2005). PIEZO2, whose non-cell-autonomous skeletal role *via* proprioceptive neurons is discussed in Section 2.1, fits the pattern in a distinct sense: the skeletal phenotype of proprioceptor-specific Piezo2 deletion emerges postnatally and progresses with mechanical loading rather than during embryonic morphogenesis (Assaraf et al., 2020). A common structural feature unites these cases: the mechanosensor is present, transcriptionally regulated during relevant cell fate decisions, often biochemically active in cell culture, and yet dispensable under the mechanical conditions in which standard phenotypic evaluations are performed.

Three interpretations of these observations dominate the literature. The first invokes functional redundancy among mechanosensor families, reasoning that co-expressed channels compensate for the loss of any individual member. The second invokes developmental compensation—upregulation of alternative channels or pathways during embryogenesis—to explain the absence of an overt phenotype. The third, most pessimistic, concludes that the channel in question is not in fact a *bona fide* mechanosensor in the tissue of interest. Each interpretation captures part of the biology, and each has explanatory force in specific cases. But each is also incomplete in an important respect. Redundancy claims are rarely tested by compound knockouts, leaving them speculative rather than demonstrated; compensation claims are seldom accompanied by the transcriptomic or functional evidence of substitute pathway engagement that would make them rigorous; and the “not-really-a-mechanosensor” interpretation fails to explain the evolutionary conservation, lineage-regulated expression, and biochemical force-responsiveness that these channels routinely display.

Before proposing a new framework it is necessary to ask whether the three established interpretations already account for the pattern assembled above. We consider each in turn, and argue that each captures part of the biology but leaves a specific and consequential gap.

#### 5.1.1 Functional redundancy

The redundancy interpretation holds that co-expressed mechanosensors compensate for one another, so that the loss of any single channel is buffered by its neighbors. This is a reasonable expectation—mechanosensor families are indeed co-expressed in most tissues—and it is almost certainly correct in some cases. Its limitation is evidentiary rather than conceptual: redundancy is rarely demonstrated. A genuine test requires compound loss-of-function—simultaneous deletion of the candidate and its proposed backups—followed by recovery of the predicted phenotype, and such compound-knockout experiments are seldom performed for mechanosensitive channels. As invoked in most of the cases above, redundancy is therefore an inference from a negative result rather than a positive finding. Moreover, redundancy in its simple form predicts that the phenotype, once unmasked by compound deletion, should appear under the same standard conditions in which the single knockouts were evaluated. It does not naturally predict that a single-channel phenotype should appear specifically and only under an altered mechanical regime, which is what the cases above display.

#### 5.1.2 Developmental compensation

The developmental-compensation interpretation holds that the absence of an overt phenotype reflects the active upregulation of alternative channels or pathways during embryogenesis. This is a stronger claim than redundancy because it specifies a mechanism—substitution—and it is supported in some developmental systems. Its limitation, again, is that the supporting evidence is usually not supplied: a rigorous compensation claim requires transcriptomic or functional demonstration that the substitute pathway is in fact engaged, and such evidence rarely accompanies the knockout result it is invoked to explain. Compensation also shares with redundancy a directional prediction that does not fit the data well. If a compensatory program operates during development, the developmental phenotype is masked, but there is no general reason for the compensation to fail selectively under regenerative or pathological mechanical loading; compensation predicts a quietly normal animal, not one that is normal until its tissue is mechanically challenged.

#### 5.1.3 The ‘not-a-mechanosensor’ interpretation

The most deflationary interpretation concludes that a channel producing no knockout phenotype is simply not a *bona fide* mechanosensor in the tissue of interest, and that its *in vitro* force-responsiveness is an artifact of overexpression or non-physiological stimulation. For some channels this may be correct, and it should not be dismissed. But applied across the pattern above it fails to account for features that the same sections document: the evolutionary conservation of these channels, their lineage-regulated expression during the relevant cell fate decisions, and their reproducible biochemical responsiveness to force. An interpretation that must treat all three of these as coincidental is doing less explanatory work than one that accommodates them.

#### 5.1.4 What the alternatives share, and what is missing

The three interpretations are not mutually exclusive, and we do not argue that any is false; each is likely correct for some channels. Our point is that all three share an unexamined assumption—that a biologically important mechanosensor should reveal itself under the standard evaluation regime of normal development, standard husbandry, and homeostatic or narrowly circumscribed challenge. That assumption is well founded for genes required continuously or for morphogenesis. It is not well founded for a gene whose function is engaged only under a mechanical regime that the standard pipeline never imposes. None of the three established interpretations predicts the specific signature seen in the cases above: a phenotype that is absent in development and homeostasis but emerges under mechanical perturbation—altered matrix composition, injury-altered loading, pathological stiffening or softening—rather than under arbitrary metabolic or genetic stress. It is this mechanical specificity that the existing interpretations leave unaddressed, and it is precisely this gap that the Regeneration-Specific Mechanosensor hypothesis is formulated to fill. The RSM framework is therefore best understood not as a competitor to redundancy and compensation but as the missing fourth interpretation—the one that takes the conditional, mechanically specific nature of the phenotype as the primary datum rather than as noise.

Two clarifications are important before developing the interpretive framework. First, the knockout paradox as we formulate it does not apply to mechanosensors whose loss is embryonic lethal—PIEZO1 in vascular endothelium, YAP/TAZ in multiple lineages, talin, vinculin, and FAK are all developmentally essential, and their essentiality is evident under standard evaluation (Table 1). The paradox concerns specifically the subset of mechanosensors whose constitutive knockout is compatible with apparently normal development and homeostasis, despite molecular, transcriptomic, and biochemical evidence of relevance to mechanotransduction. Second, the paradox is not equivalent to the classical concept of conditional lethality or synthetic phenotype in genetics, though it is formally related. What distinguishes the mechanosensor case is the specific nature of the conditional challenge: the phenotype, when it emerges, is unmasked under a mechanical perturbation—altered matrix composition, injury-altered loading geometry, pathological stiffening or softening of the pericellular environment—rather than under arbitrary physiological, metabolic, or combinatorial genetic stress. This mechanical specificity is what licenses the interpretive framework developed in the next section, in which we propose that the silent mechanosensors of development are deployed as the active mechanosensors of regeneration. The developmental-versus-regenerative contrast at issue here is encoded directly in Table 1, whose Dev. KO and Regen. KO columns catalog, for every mechanosensor reviewed, the discordance between morphogenetic dispensability and regenerative requirement; the reader is referred to that table as direct evidence for the pattern this section describes.

**TABLE 1:** Comparative catalog of major mechanosensors.

Mechanosensor	Family	Architecture	Stimulus	Tissues	Dev. KO	Regen. KO	Key reference
PIEZO1	PIEZO	Trimeric blade	Membrane tension, shear	Endothelium, RBC, bone, urothelium	Embryonic lethal (vascular)	Required (vascular repair)	Coste et al., 2010; Ranade et al., 2014
PIEZO2	PIEZO	Trimeric blade	Touch, proprioception, stretch	Sensory neurons, Merkel, proprioceptors	Mild developmental	Required (skeletal integrity *via* proprioception)	Assaraf et al. (2020)
TRPV4	TRP	Tetrameric	Hypotonicity, compression	Cartilage, endothelium, lung	Mild	Likely required	O'Conor et al. (2014)
TRPA1	TRP	Tetrameric	Stretch, irritants	Sensory, chondrocytes	Normal skeleton	Predicted required (RSM)	Horváth et al. (2016)
TRPM7	TRP	Tetrameric + kinase	Mg^2+^/Ca^2+^ flux, shear	Broad	Embryonic lethal	Not characterized	Schmitz et al. (2003)
TRPC1	TRP	Tetrameric	Stretch (debated)	Smooth muscle, skeletal muscle	Mild	Not characterized	Maroto et al. (2005)
TMC1	TMC	Dimeric	Tip-link tension, lipid	Hair cells, MSC (emerging)	Deafness	Not characterized	Pan et al., 2013; Jeong et al., 2022
TMC2	TMC	Dimeric	Tip-link tension	Hair cells (transient)	Mild	Not characterized	Kawashima et al. (2011)
YAP/TAZ	Transcr. Coact.	Cytosolic→nuclear	Stiffness, geometry	Universal	Embryonic lethal	Required (multiple tissues)	Dupont et al. (2011)
Talin	Adhesion	Cytoplasmic rod	Force-induced unfolding	Universal	Embryonic lethal	Required	del Rio et al. (2009)
Vinculin	Adhesion	Helical bundle	Talin-recruited under load	Universal	Embryonic lethal	Required	Grashoff et al. (2010)
FAK	Adhesion kinase	Cytoplasmic kinase	Adhesion maturation	Universal	Embryonic lethal	Required	Schaller (2010)
α-Catenin	Cadherin compl.	Cytoplasmic	Junctional tension	Epithelia, endothelia	Embryonic lethal	Required	Yonemura et al. (2010)
E-cadherin	Cadherin	Transmembrane	Cell–cell tension	Epithelia	Embryonic lethal	Required	Borghi et al. (2012)
RhoA	Rho GTPase	Small GTPase (GTP/GDP switch)	Integrin engagement, adhesion maturation	Universal	Embryonic lethal (vascular)	Required (wound repair, MSC commitment)	Huang and Ingber (2005)
ROCK	Rho effector kinase	Ser/Thr kinase	RhoA-GTP binding	Universal	ROCK1+2 embryonic lethal	Required (MSC osteogenic commitment)	Arnsdorf et al. (2009)
Non-muscle myosin II	Molecular motor	Hexameric (heavy/light chains)	MLC phosphorylation	Universal	MYH9 embryonic lethal	Required (force generation, wound contraction)	Yamada et al. (2023)
MLCK	Ca^2+^/CaM kinase	Ca^2+^/calmodulin-dependent kinase	Ca^2+^ flux	Smooth muscle/broad	Tissue-specific viable	Required (PSC lineage specification)	Fort et al. (2026)
α-Actinin	Actin crosslinker	Antiparallel dimer	F-actin/stress fiber assembly	Universal	Isoform-dependent	Required (focal adhesion stability)	Katsuta et al. (2024)
Lamin A/C	Nuclear lamina	Intermediate filament	Nuclear deformation	Most adult tissues	Postnatal lethal	Required	Swift et al. (2013)
LINC complex	Nuclear envelope	SUN/KASH	Cyto-nuclear coupling	Universal	Variable	Required (*in vivo*)	Lombardi et al. (2011)
KLF2	Transcr. factor	Zn-finger TF	Endothelial shear	Endothelium	Embryonic lethal (vascular)	Required (valve)	Lee et al. (2006)
COCH	ECM	Secreted	Inner ear/emerging	Cochlea, MSC (emerging)	Late hearing loss	Not characterized	Robertson et al. (1998)

Abbreviations: RBC, red blood cell; MSC, mesenchymal stem cell; KO, knockout; RSM, regeneration-specific mechanosensor; transcr., transcriptional; coact., coactivator; compl., complex; TF, transcription factor; MLC, myosin light chain; CaM, calmodulin; PSC, pluripotent stem cell.

### 5.2 The RSM hypothesis

**FIGURE 3:**
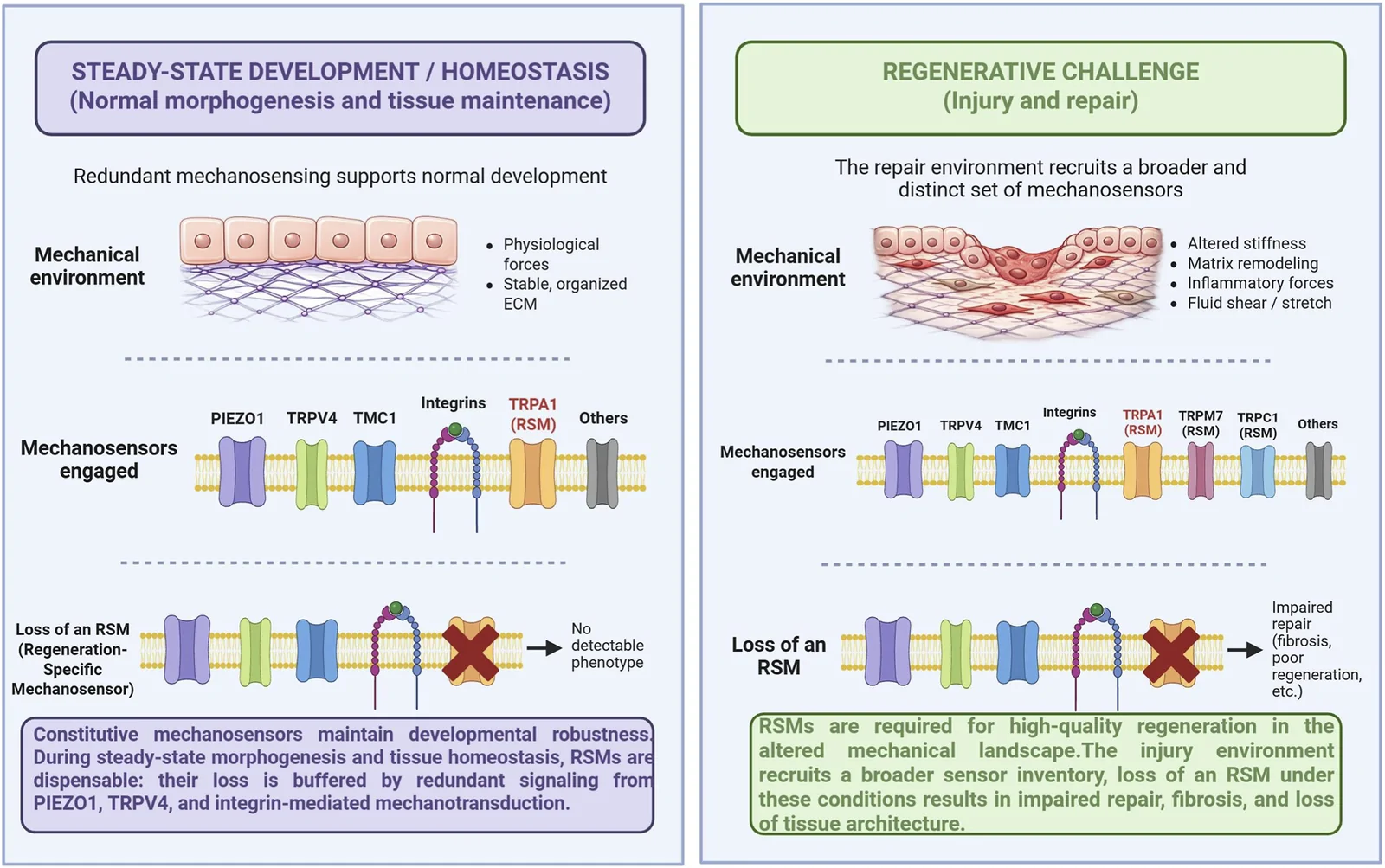
Developmental *versus* regenerative mechanotransduction. Schematic representation of the Regeneration-Specific Mechanosensor (RSM) hypothesis. Left: under steady-state developmental and homeostatic conditions, redundant mechanosensing supports normal morphogenesis, and loss of any single RSM (TRPA1 illustrated) produces no detectable phenotype. Right: under regenerative challenge, the altered mechanical landscape of the repair environment recruits a broader and distinct set of mechanosensors; loss of an RSM in this regime produces a substantial defect in repair quality. The framework predicts that several mechanosensors currently regarded as functionally redundant or dispensable will be revealed as essential when evaluated under appropriate regenerative challenge.

We propose that a defined subset of mechanosensors is dispensable for morphogenesis but essential for regeneration. Under this framework, regeneration-specific mechanosensors (RSMs) are deployed selectively when a tissue must reorganize *de novo* against an altered mechanical landscape—following injury, inflammation, or pathological remodeling—but are not engaged during the steady-state mechanical regime of normal development or adult homeostasis. In the developmental regime, redundant force-sensing mechanisms are sufficient to support morphogenesis, and the loss of any single channel produces a mild or undetectable phenotype. In the regenerative regime, the mechanical landscape is fundamentally altered: matrix composition, stiffness, strain magnitudes, and loading frequencies all deviate from their physiological setpoints, and a different and broader set of mechanosensors is recruited to interpret this novel environment. Channels that were silent during morphogenesis become essential during repair (Figure 3).

The RSM hypothesis must be distinguished from three interpretations of the knockout paradox that were catalogued across Sections 2–4 and consolidated at the close of Section 4.6 — redundancy, developmental compensation, and context-dependent mechanotransduction. Each is a serious alternative, and we consider them in turn.

#### 5.2.1 Redundancy

The standard explanation for mild single-channel knockout phenotypes is that co-expressed mechanosensors share substrates and effectors, so the loss of any one channel is buffered by others. Redundancy is real and demonstrably explains some cases. But strict redundancy struggles to rationalize the evolutionary maintenance of distinct channel families with non-overlapping gating modalities, expression dynamics, and chemical agonist profiles. If TRPA1 in cartilage were functionally redundant with TRPV4 or PIEZO1, the conservation of three distinct sensing modalities—chemical, osmotic, and tension—over hundreds of millions of years of vertebrate evolution would be difficult to explain. Under the RSM hypothesis, these channels are not redundant; they are deployed in different mechanical regimes, and the developmental window does not engage the regenerative subset.

#### 5.2.2 Developmental compensation

Compensation differs from redundancy in invoking an active response to gene loss—rewiring of pathways or recruitment of alternative cell populations during embryogenesis—that masks the role of the deleted channel. It is well established that constitutive knockouts can permit compensatory programs that conditional or inducible deletion does not (El-Brolosy and Stainier, 2017). RSM and compensation predict opposite outcomes in a specific experiment: conditional inactivation of an RSM timed to the regenerative window. Under compensation, the program installed during development would persist and continue to buffer the loss; under RSM, the channel was never required during development, and its targeted loss in the regenerative window should produce a discrete repair defect.

#### 5.2.3 Context-dependent mechanotransduction

The most subtle alternative posits that mechanosensors are constitutively active and that their downstream consequences are modulated by cellular state, matrix environment, or co-active signaling. The context-dependence of YAP/TAZ activation across lineages—driving proliferation in some, growth arrest or apoptosis in others—is a well-characterized example (Dupont et al., 2011). Under this framing, TRPA1 would be engaged in chondrocytes at all times, with its downstream readout varying between developmental, homeostatic, and regenerative contexts. The RSM hypothesis makes a stronger and more specific claim: the channel is not engaged during morphogenesis, regardless of context, and is recruited only when a regenerative challenge alters the mechanical environment beyond a threshold. The two views diverge on a measurable variable—basal channel activity in developing *versus* regenerating tissue. Under context-dependent mechanotransduction, basal activity should be detectable at all times and only its downstream effects should vary; under RSM, basal activity itself should be low or absent in the developmental window and rise upon injury. Patch-clamp recordings from staged tissue, or genetically encoded activity reporters expressed across developmental and regenerative time, can adjudicate between the two interpretations.

These three alternatives are not mutually exclusive, and elements of each plausibly apply to particular mechanosensors. The RSM hypothesis does not displace them. It identifies a specific subset of mechanosensors—transcriptionally regulated, pharmacologically tractable, evolutionarily conserved, yet phenotypically silent under constitutive knockout—whose behavior is better explained by regeneration-specific deployment than by any of the three alternatives alone. The three predictions that follow operationalize this distinction.

The RSM hypothesis makes three concrete predictions that are testable with existing experimental tools. First, RSM expression should be low or absent in homeostatic adult tissue but inducibly upregulated following injury, with kinetics that parallel the time course of repair. Second, conditional or inducible loss-of-function of RSMs in the regenerative window—but not in the developmental or homeostatic windows—should produce defects in tissue repair quality. Third, pharmacological activation of RSMs during the regenerative window should enhance repair quality, while activation during the homeostatic window should be neutral or, depending on the channel, mildly disruptive. These three predictions operationalize the distinctions drawn above and define a clear experimental program for testing the RSM framework.

### 5.3 TRPA1 as the prototypical RSM

TRPA1 in cartilage provides the prototypical case for the RSM framework. The published phenotype of Trpa1-null mice is consistent with each of the three RSM predictions. Skeletal development is normal, indicating dispensability during morphogenesis. The MIA-induced osteoarthritis phenotype is unchanged from wild-type, consistent with the interpretation that the MIA model—which produces rapid chemical destruction of articular cartilage rather than a regenerative challenge—does not engage TRPA1’s regeneration-specific function (Horváth et al., 2016). The model therefore generates the loss-of-tissue without generating the regenerative attempt that would, on the RSM hypothesis, recruit TRPA1.

By contrast, the RSM hypothesis predicts that TRPA1 should be engaged in cartilage repair models that involve genuine regenerative attempts: full-thickness osteochondral defects, microfracture-stimulated repair, mesenchymal stem cell–based regenerative therapies, and *de novo* cartilage formation in tissue-engineered constructs. To our knowledge, the majority of these experimental settings have not yet been tested under conditional Trpa1 loss-of-function, and the field has therefore overlooked a mechanistically central player in regenerative cartilage biology. Our prediction is that conditional Trpa1 deletion in mesenchymal progenitors during a regenerative challenge will produce a substantial repair-quality defect, with disorganized matrix architecture and impaired columnar organization—phenotypes invisible in either steady-state development or the MIA model.

### 5.4 Generalizing the RSM hypothesis

The RSM framework generates testable predictions for several other mechanosensors. PIEZO2 is a strong candidate, though the regeneration-relevant tissue is skeletal rather than muscular. Conditional deletion of Piezo2 in chondrogenic or osteogenic lineages (Col2a1-Cre, Prx1-Cre, Col1a1-Cre) produces only mild skeletal phenotypes and does not recapitulate the abnormalities observed in human PIEZO2 loss-of-function, indicating dispensability during skeletal morphogenesis. By contrast, deletion in proprioceptive neurons using the PValb-Cre driver produces progressive postnatal spinal malalignment and hip dysplasia that phenocopy the human syndromes, revealing a non-cell-autonomous mechanism by which proprioceptive mechanosensing regulates skeletal integrity against the mechanical loads of postnatal growth (Assaraf et al., 2020). This developmental mildness combined with postnatal essentiality for tissue-scale structural maintenance fits the RSM framework in spirit if not in the strict injury-repair sense: the channel is dispensable during morphogenesis but becomes essential for the ongoing mechanical homeostasis that sustains skeletal form thereafter. TRPV4 represents an intermediate case: it is clearly required for postnatal cartilage homeostasis (O’Conor et al., 2014) but may also play a regeneration-specific role in cartilage repair, distinct from its homeostatic function. Members of the TMC family expressed outside the auditory system are candidates whose biology is sufficiently early-stage that the RSM framework provides useful experimental scaffolding for prioritizing future loss-of-function experiments.

Beyond ion channels, the RSM framework may apply to mechanosensitive components of focal adhesions and to nuclear mechanotransducers whose homeostatic function appears redundant but whose regenerative role has not been systematically tested. The framework is general; the specific molecular candidates are diverse.

### 5.5 Experimental implications

If the RSM hypothesis is correct, the field’s standard approach to evaluating mechanosensor function—constitutive knockout in development, evaluation of homeostatic phenotype in adulthood—is structurally biased toward false negatives. The corollary is that several published “negative” results may not in fact establish biological irrelevance, but rather reflect a mismatch between the experimental regime and the regime in which the channel is biologically essential. The RSM framework therefore suggests a broadened experimental standard: mechanosensors of unclear function should be evaluated not only in development and homeostasis, but explicitly in regeneration, with conditional and inducible loss-of-function tools deployed in injury models and in *de novo* tissue formation systems. We anticipate that adoption of this broadened standard will reveal essential roles for a substantial fraction of currently understudied mechanosensors, and we anticipate that the resulting expanded inventory of regeneration-specific targets will substantially enlarge the translational opportunity space outlined in Section 6.

## 6 Therapeutic implications and translational outlook

Mechanobiology is no longer a purely academic discipline. The maturation of the molecular toolkit, the recognition that engineered mechanical environments can direct stem cell fate with morphogen-comparable specificity, and the emergence of regeneration-specific mechanosensitive targets together constitute a translational opportunity space of substantial magnitude. We organize this section around five themes, beginning with the cardiovascular system as the most clinically advanced application area.

### 6.1 Mechanosensitive targets in cardiovascular medicine

The cardiovascular system contains the most clinically advanced examples of mechanosensitive drug targets. Yoda1, the small-molecule PIEZO1 agonist, has been explored experimentally for the modulation of vascular tone, lymphatic function, and erythrocyte volume regulation, and provides a chemical tool for testing whether PIEZO1 activation can be therapeutically harnessed in vascular pathology. Yoda1 is, however, a first-generation tool compound whose limited aqueous solubility and modest potency constrain its physiological and translational use, and PIEZO1 pharmacology has since advanced through structure–activity-guided derivatives of the Yoda1 scaffold. Yoda2, generated by 4-benzoic acid modification of Yoda1, offers improved efficacy, potency, and solubility, making it a more robust activator for physiological assays and a better starting point for drug-like optimization (Parsonage et al., 2023). Yaddle1, in which the central thiadiazole ring is replaced by an oxadiazole bearing a trifluoromethyl substituent, similarly improves potency and aqueous solubility relative to Yoda1, and has been characterized not only as a chemical activator but as a vaccine adjuvant that enhances T-cell activation—extending mechanosensitive pharmacology beyond the cardiovascular system into immune modulation (Goon et al., 2024). Together these next-generation agonists illustrate that PIEZO1 is becoming a genuinely druggable target rather than a channel accessible only to a single laboratory probe, and that the therapeutic reach of mechanosensitive activation now spans vascular, hematopoietic, and immunological applications. GsMTx4 and related peptidic inhibitors of cationic mechanosensitive channels are under investigation for conditions of pathological mechanical loading, including muscular dystrophy and arrhythmia substrates. TRPV4 antagonists have advanced into clinical trials for pulmonary hypertension and pulmonary edema, where TRPV4-dependent endothelial responses to mechanical wall stress contribute to disease progression.

Beyond pharmacology, the recognition that flow-responsive signaling is central to atherosclerosis and aortic aneurysm pathogenesis has motivated a generation of work on hemodynamic biomarkers and on engineered vascular conduits whose mechanical compliance is matched to native vessels. From the fetal cardiology perspective, the prospect of mechanosensitive intervention is particularly intriguing: early-life cardiovascular pathology, including hypoplastic left heart syndrome and twin–twin transfusion-related cardiomyopathy, are mechanically driven processes in which timely modulation of intracardiac flow or wall mechanics could in principle redirect downstream developmental trajectories. The bridge between fetal and adult cardiology is mechanobiological, and pharmacological tools that act on flow-responsive endothelial signaling will increasingly span both domains.

### 6.2 Engineered niches for regeneration

The deliberate engineering of mechanical microenvironments for regenerative therapy is among the most promising near-term translational applications of mechanobiology. Tunable hydrogels with stiffness gradients, anisotropic fiber alignment, and dynamic stiffening capacity now permit the construction of regenerative niches whose mechanical properties match the target tissue and evolve appropriately during repair. Bioprinted constructs incorporating multiple cell types within mechanically defined matrices are advancing toward clinical evaluation in cartilage repair, vascular grafting, and skin regeneration. Organ-on-chip systems incorporating physiologically relevant flow and strain provide platforms not only for drug discovery but for the optimization of cellular therapies under mechanically realistic conditions.

### 6.3 Stem cell therapy mechanics

The mechanical environment encountered by therapeutic stem cells after transplantation is increasingly recognized as a determinant of graft survival and functional integration. Cells optimized *in vitro* on stiff plastic substrates frequently behave differently after injection into a soft host tissue, and this mismatch is one of the underappreciated causes of cell therapy failure in regenerative medicine trials. Pre-conditioning cells in mechanically appropriate niches before transplantation, encapsulating cells in mechanically tuned carriers, and selecting cell preparations enriched for mechanosensor expression are emerging strategies for improving therapeutic outcomes.

### 6.4 Mechanobiology in disease beyond cardiovascular

Mechanobiological dysregulation contributes to disease across many organ systems. In osteoarthritis, the loss of TRPV4-dependent anabolic responses to compressive loading parallels the loss of cartilage matrix integrity, and pharmacological restoration of mechanosensitive signaling represents an emerging therapeutic strategy. In organ fibrosis, mechanochemical feedback loops involving YAP/TAZ and matrix stiffening produce a self-reinforcing pathology that has motivated the development of pathway inhibitors now entering clinical evaluation. In metastatic cancer, the mechanical properties of the stromal environment direct the invasive behavior of tumor cells and influence the homing of metastatic seeds, with implications for both diagnostic stratification and therapeutic targeting. In muscular dystrophy and other heritable myopathies, defects in mechanosensitive signaling networks downstream of dystrophin-associated complexes contribute to disease progression and represent emerging therapeutic targets.

### 6.5 Translation challenges

The translation of mechanobiological insights into clinical therapy faces several structural challenges. Drug development pipelines for mechanosensitive targets require new categories of pharmacological tools—channel modulators that act in physiologically relevant force ranges, lipid-modulating compounds that act at the protein–lipid interface, and biological agents that engineer the mechanical microenvironment of the target tissue. Biomarkers for mechanobiological disease and for mechanosensitive drug response are immature, and the regulatory pathways for mechanically engineered devices and combination products are evolving. The Regeneration-Specific Mechanosensor framework developed in Section 5 has direct translational relevance: it predicts that the highest-value mechanosensitive drug targets are not necessarily those active in homeostasis, but those selectively active during repair, where pharmacological enhancement can increase regenerative capacity without disrupting steady-state physiology. This selectivity, if borne out experimentally, will substantially improve the therapeutic index of mechanosensitive interventions and should be a priority for translational programs in cartilage, muscle, and cardiovascular regeneration over the coming decade.

## 7 Future perspectives

### 7.1 Open questions

Three sets of open questions appear to us most consequential for the field’s progress. First, what determines the hierarchy of mechanosensors during lineage commitment? In any given commitment decision, multiple mechanosensitive channels and adhesion complexes are co-expressed, and the principles by which their inputs are weighted, integrated, or rendered mutually exclusive remain poorly understood. Second, what is the mechanical logic of multicellular symmetry breaking? While individual examples have been characterized in detail, a general theory of how mechanical fields participate in the spontaneous emergence of polarity in cell aggregates is still being constructed, and quantitative experimental systems for testing such a theory are only now becoming available. Third, how general is the developmental–regenerative distinction articulated by the Regeneration-Specific Mechanosensor hypothesis? The TRPA1 case is suggestive but a single example is not a generalization, and the field will benefit from systematic tests of the RSM hypothesis across multiple channel families and tissue systems.

### 7.2 Technological frontiers

Several technological developments are likely to substantially enlarge the experimental access of the field over the coming years. Spatial transcriptomic platforms with subcellular resolution will permit direct mapping of mechanosensor expression onto spatially defined mechanical microenvironments, providing the experimental substrate for asking where in a tissue each mechanosensor is engaged and under what mechanical conditions. Brillouin microscopy permits non-invasive measurement of tissue mechanical properties at micron resolution in living samples, removing one of the most persistent technical barriers to *in vivo* mechanobiology. Genetically encoded FRET tension sensors targeted to specific cytoskeletal and adhesion proteins permit real-time visualization of force transmission within living cells. Instrumented organoid systems that combine controlled mechanical inputs with high-content readouts of cell fate are emerging as a new experimental platform with substantial discovery potential. All-atom molecular dynamics simulations of mechanosensitive channel gating, increasingly feasible at physiologically relevant timescales, are beginning to provide atomistic context for the experimental observations and to identify candidate druggable interfaces.

### 7.3 Cross-disciplinary synthesis

Mechanobiology is intrinsically interdisciplinary. Its progress depends on continuing collaboration among biophysics, structural and computational chemistry, developmental and regenerative biology, tissue engineering, and clinical medicine. The most productive groups in the field are those that span at least two of these disciplines structurally rather than collaboratively, and the most productive papers are those in which the molecular, cellular, and tissue scales are addressed in a single integrated framework. We have organized the present review around such an integration, and we close with the observation that the field’s next decade will likely be characterized by the increasing adoption of unifying frameworks—of which the Regeneration-Specific Mechanosensor hypothesis is one candidate among several—that permit the diverse experimental observations of cellular and tissue mechanobiology to be organized into a coherent and testable theoretical structure.

## Data Availability

The datasets presented in this study can be found in online repositories. The names of the repository and accession numbers can be found below: NCBI Gene Expression Omnibus (GEO), GSE325268 and GSE325419.
